# Advancing the application of systems thinking in health: managing rural China health system development in complex and dynamic contexts

**DOI:** 10.1186/1478-4505-12-44

**Published:** 2014-08-26

**Authors:** Xiulan Zhang, Gerald Bloom, Xiaoxin Xu, Lin Chen, Xiaoyun Liang, Sara J Wolcott

**Affiliations:** School of Social Development and Public Policy (SSDPP), Beijing Normal University, 19 Xinjiekouwai Street, Beijing, 100875 China; Institute of Development Studies (IDS), the University of Sussex, Sussex, Brighton BN1 9RE UK

**Keywords:** Health system, Institutional development, Rural health finance, System resilience

## Abstract

**Background:**

This paper explores the evolution of schemes for rural finance in China as a case study of the long and complex process of health system development. It argues that the evolution of these schemes has been the outcome of the response of a large number of agents to a rapidly changing context and of efforts by the government to influence this adaptation process and achieve public health goals.

**Methods:**

The study draws on several sources of data including a review of official policy documents and academic papers and in-depth interviews with key policy actors at national level and at a sample of localities.

**Results:**

The study identifies three major transition points associated with changes in broad development strategy and demonstrates how the adaptation of large numbers of actors to these contextual changes had a major impact on the performance of the health system. Further, it documents how the Ministry of Health viewed its role as both an advocate for the interests of health facilities and health workers and as the agency responsible for ensuring that government health system objectives were met. It is argued that a major reason for the resilience of the health system and its ability to adapt to rapid economic and institutional change was the ability of the Ministry to provide overall strategy leadership. Additionally, it postulates that a number of interest groups have emerged, which now also seek to influence the pathway of health system development.

**Conclusions:**

This history illustrates the complex and political nature of the management of health system development and reform. The paper concludes that governments will need to increase their capacity to analyze the health sector as a complex system and to manage change processes.

## Background

China created a system of rural health care finance during the period of the socialist planned economy (1949 to 1978), when the level of economic development was very low, and a large proportion of the population lived in poverty. The Cooperative Medical System (CMS) provided basic health benefits to most rural farmers. By 1976, more than 90% of rural villages had a CMS and a network of preventive and curative health facilities at county, township, and village levels. Most services were provided by “barefoot doctors”, who had limited training but provided timely and low cost treatment to rural residents [1]. They were supervised by medical doctors and they could refer patients to county hospitals. This rural health system contributed to significant improvements in access to basic health services and in health outcomes [2]. This rural health finance system collapsed during China’s transition to a market economy, which began in 1978. During the 1980s and 1990s, efforts were made to rebuild it, with little success. However, starting in 2002, the government began to implement so-called New Cooperative Medical Schemes (NCMS) and, by 2012, 805 million rural residents, or 98% of the rural population, were covered by NCMS [3].

This paper explores the evolution of CMS and NCMS since the late 1970s as a case study of the long and complex process of health system development. It argues that its trajectory has been the result of the response of large numbers of agents to a rapidly changing context and of efforts by the government to influence this adaptation process. It draws on the concept of resilience, which Walk et al. [4] define as “*the capacity of a system to absorb disturbance and reorganize while undergoing change so as to still retain essentially the same function, structure, identity, and feedbacks*”. It concludes that the way that the government manages the adaptation of the health system to rapid change strongly influences the degree to which it meets socially agreed objectives of providing access to safe and effective services for all [5].

### Applying system thinking to health care systems: a literature review

#### Understanding health care systems as complex adaptive systems

There is a growing interest in applying concepts of complex adaptive systems (CAS) [6–14] to the analysis of health systems. A CAS has many components, often called agents, which interact in apparently random ways [15]. Through these interactions, patterns emerge and the system is continually self-organizing through processes of emergence and feedback. Agents in the system are ignorant of the behavior of the system as a whole, responding only to local information [16–19]. Policy-makers, who want to implement a new policy and overcome resistance [13], need to pay attention to the context, the behavior and networks of agents, and likely feedback loops [20].

A number of case studies in the advanced market economies have applied CAS concepts to different aspects of health system performance, such as disease control [21], nursing homes [22], palliative care [23], family practices [24], and primary care [25–28], in designing evaluation research [29–33], in interpreting research findings [34, 35], and in other health system areas [36–39]. These studies have increased our understanding of the role and behavior of agents involved in health systems.

There are a growing number of studies of health systems in low- and middle-income countries. Many of these countries have weak institutional arrangements in comparison to the advanced market economies and this influences how policy is translated into changes in system behavior [40]. Xiao et al. [41] explore this with regard to China’s implementation of an essential drugs policy. They demonstrate that the interaction of responses by different actors has led to divergent and unpredicted outcomes. They conclude that the management of policy change in rural China needs to take into account the emergence of adaptive and self-organized behavior and that many changes are nonlinear.

Paina and Peters [42] offer a macro perspective on strategies for taking health system changes to scale. They examine the impact of system history, context, and political and institutional structures on the complex pathways of change. The interactions of system components and phased change and transitions are important aspects of successful scaling up of health services. The authors conclude with a call for more research on the management of health system transition and adaptation to changing contexts.

#### The long, dynamic, and complex process of health system development

It is important to understand the performance of a health system in its broader context. This is especially important in countries, like China, which are in the midst of a number of rapid and interconnected changes. Work on health system development can draw on a substantial body of work, which applies the lens of resilience thinking and CAS to studies of changes in social-ecological systems. Resilience thinking offers a good framework for examining the long, dynamic, and complex process of system change. Folke et al. [5] argue that adaptation and transformation are essential for maintaining system resilience. They view adaptability as the capacity of actors in a system to influence resilience and transformability as the capacity to create a fundamentally new system. They differentiate between two types of transformation. Forced transformation happens at a scale beyond the influence of local actors and is imposed by external forces. Another type of transformation is the deliberately initiated transformational process by people involved at multiple scales and can lead to feedback effects that conclude in whole system change.

The literature on adaptive management and transition management has roots in CAS theory [5]. Adaptive management is concerned with the establishment of a continuous learning process that responds to new information by reformulating hypotheses and models, and understanding policy implementation as experiments. Transition management is concerned with the dynamics of structural change of the system. There is a spectrum between adaptability and transformability from the lens of resilience. Identifying the key transition points and understanding the mechanisms of how system transformations are initiated, facilitated, and influenced can help us understand the health system development process.

Gell-Mann argues that it is important to differentiate between what is adaptive and the outcome of a process of adaptation [17], he maintains that the latter can be maladaptive due to the influence of selection pressures. From a system evolution perspective, it is crucial to understand the impact of individual adaptations for system goal achievements, system resilience, and choices of system transition [43]. The mal-adaption is similar to policy resistance discussed by Tan et al. [14]. This paper applies these concepts to the analysis of the pathway of development of China’s NCMS system over three and a half decades. It explores what has triggered transitions and how adaptation by agents has played an important role in driving change. It also explores how the Ministry of Health has attempted to maintain system resilience by enabling agents to adapt to a changing macroeconomic context while acting to ensure that the system maintained its function and achieved agreed policy goals.

## Methods

This study is a retrospective review of the development of the rural health system over three and half decades. We decided to focus on NCMS for several reasons. First, the authors have already undertaken research on NCMS, and several participated in the implementation process or served as advisors to government. Second, the authors have access to key informants who were involved in the policy process. Third, the first author is on the National Healthcare Reform Expert Committee. This committee includes representatives of all relevant government agencies including the State Development and Reform Commission, the Ministries of Health, Finance, Civil Affairs (for the poor), Human Resources and Social Insurance, and so forth.

An insider’s perspective has strengths and limitations. Being part of the change process provides information on the “black box” of policy negotiation and decision-making, and the core issues being debated. It can also provide insights on the thinking of top decision-makers when they were in the conference rooms. However, these impressions might not be accurate, and they may be influenced by a number of biases. Realizing the pros and cons of the core data sources, this case study selected supplementary sources of data to minimize the potential risk of bias.

We approached the former Minister of Health whose tenure ended in 2003. We asked him to identify key informants who had gone through the longest period of NCMS development. We identified four key informants including the former minister, the rural health bureau director, the NCMS office director, and the former Shan’xi province Director of Health Bureau. We asked the four key informants to define stages of the development of NCMS from their own perspective, and identify the key “transition points” for system change. The four informants provided similar answers which were based on the changes of the national agenda for development. Based on the key informants, we built a detailed picture of NCMS system development stages and transitions.

The key informant interviews pointed to the close relationship between health system change and the national development process, and national development priorities. To use the language of CAS, the context plays a key role in driving system transitions. To test this hypothesis, we began to collect and review all national policy documents on CMS and NCMS. China has issued 92 national policies addressing rural health systems. To better understand the national development priorities, we reviewed the memoirs of the former premier Zhu Rongji, and interviewed the former health minister on his reflections of the national policy priorities.

We paid particular attention to the policy environment and public policy priorities at each “transition point” to understand the interplay between health policy and national development agenda transitions. We developed a semi-structured questionnaire to conduct a second round of interviews of the key informants on the processes of multi-agency bargaining and negotiation, and to identify the key determinants for making the “transition” happen. The data on adaptation mainly came from three sources. First, we interviewed the NCMS office director, two provincial (Shan’xi and Hubei) health bureau directors, and three county health bureau directors to learn the process of the NCMS policy formations and implementation. We also reviewed internal reports on NCMS, collected statistics of rural health system elements based on the government health information system, and the policies issued by the national government and the health ministry on NCMS. The third source is the large body of published literature on NCMS. All interviews took place between January 2013 and January 2014. Finally, to understand the status changes of NCMS, we traced the policy documents on rural China development. Each January, the State Council of China issues the 1st National Policy Document, and historically, all 1st Documents concern rural development, including health, education, social security, agriculture, etc. We hoped that through this method, we would have a full picture of China’s NCMS development.

It is important to keep in mind the relatively narrow focus of our data collection on the perspectives of health sector policy actors. We have not attempted to collect information on the perspectives of providers or users of health services. Nor have we explored the points of view of senior policy actors outside the health sector. Despite these limitations, the study provides a useful insight into the challenges of managing the adaptation of a health system to a rapidly changing context, as seen by those most directly responsible for providing policy leadership.

## Results

Starting in the late 1970s, rural health finance went through three main transitions. Each was a forced transition [5], because it was largely determined by a shift in the national development agenda and in the understanding by the top leaders’ of the key issues concerning rural health system development. The adaptation processes after each transition were strongly influenced by an iteration between responses by a multitude of individual agents and government efforts to revise the rules of the game, through a series of policy initiatives. Table 1 summarizes the transition and adaption process of NMCS. Detailed analysis follows.

**Table 1:** **Transition and adaption of NCMS**

Time period	Events	Notes
1950s to 1978	The origin and development of the Cooperative Medical Scheme (CMS)	CMS: System History
Dec. 1978	China’s market oriented economic reform promoted a system of household responsibility	Transition Point I
1978 to 1979	The Ministry of Health (MoH) issued five national policies to strengthen rural health care facilities and organizations in staff compensation, training, management, investment, and subsidies to health facilities	Adaption Process I
1980s	The CMS collapsed rapidly due to lack of support from the collective economy in rural China and other reasons	
	The MoH issued many documents on rural healthcare workforce and their compensations, such as retirement and pension calculation, subsidies, private clinic permissions, support to barefoot doctors, service fee charges for immunization work done by the grassroots clinics and doctors, and service fee charges by sanitation and anti-epidemic stations	
1990s	The MoH Started to rebuild CMS, but the efforts failed for lack of consensus between different government branches and the limited investment to the rural health system	
Oct. 2002	A “*Decision on Further Strengthening the Public Health Work in Rural Areas*” was issued jointly by the Central Committee of the Communist Party of China (CPC) and the State Council in October 19, 2002. The New Cooperative Medical Scheme (NCMS) was defined as a rural cooperative medical insurance system based on a co-financing system which included volunteer contributions from individuals and financial support from central and local government	Transition Point II
2003 to 2005	NCMS pilots were carried out in approximately 300 counties in order to improve the design of reimbursement plans, the management of funding and services , etc.	Adaption Process II
2006	A large-scale interim evaluation of the scheme was carried out, which helped inform subsequent policy and promote convergence in policy design	
2006 to 2008	The expansion of NCMS in China. The coverage rate of NCMS increased from less than 10% in 2002 to more than 90% in 2008. At the same time, the number of registered doctors, assistant doctors, available beds, and inpatients treated has increased significantly in Township Health Centers and County Hospitals	
2009	The Central Committee of the CPC and the State Council jointly endorsed and issued the Guidelines on Deepening the Reform of Healthcare System after about three years of intense debate and repeated revision	Transition Point III
2009 to present	Policies on Essential Drugs, County Hospital Reforms, Payment Reforms; Integration of NCMS with Urban Health Insurance Systems at local level	Adaption Process II

### First transition and adaption to outside pressures between 1978 and 2002

The initial forced transition (from the planned economy to the market economy) took place in the late 1970s. Launched in late 1978, China’s economic reforms promoted a system of household responsibility. Land that had previously been collectively owned was allocated to individual households. The introduction of the household responsibility system was a significant contributor to the collapse of CMS, since townships could no longer allocate a share of collective production to the scheme [44]. In addition, low levels of public funding of rural health, design, and management flaws of CMS, and the lack of consensus about the future of CMS accelerated disintegration of the program. In the 1980s, CMS coverage fell to less than 10% of rural residents, with the lowest rate at 5% [2, 45, 46]. At the same time, the government ended its policy of requiring skilled health workers to remain in rural facilities. Over time, the Ministry of Health became aware of a number of problems with rural health services, such as rising costs of medical care, shortages of skilled personnel, and the resurgence of previously eradicated or controlled infectious diseases [44, 47].

The priorities of the Ministry of Health during this period were to maintain the effectiveness of the health system in meeting agreed policy goals with regard to access to services, while ensuring that health facilities remained financially viable. It pursued the latter by allowing service providers to use “market tools” to generate revenue to pay their workforce [48]. Between 1978 and 1989, the policies issued by the Ministry of Health primarily focused on maintaining the financial viability of public health organizations and ensuring that health workers were paid. From the end of 1978 to the summer of 1979, the Ministry of Health issued five national policies to strengthen rural health care facilities and organizations in staff compensation, training, management, investment, and subsidies to health facilities. More policy documents were issued between 1979 and 1988 on the rural healthcare workforce and their payment, such as retirement and pension calculation, subsidies, private clinic permissions, support to barefoot doctors, service fee charges for immunization work done by the grassroots clinics and doctors, and service fee charges by sanitation and anti-epidemic stations. By allowing the rural healthcare facilities and doctors to charge fees to maintain the delivery of health services, and by allowing the health facilities to improve their management according to the market principles and fighting for more government investment in rural health, the Ministry of Health ensured the viability of the rural health services in the face of major financial challenges [48, 49]; it did this whilst maintaining public ownership of these facilities. In addition to the public ownership of the healthcare facilities and the workforce, the professional organizations, such as the Chinese Medical Association and the Chinese Doctors’ Association, were all under the management of the Ministry of Health and helped the healthcare workers to negotiate their income, maintain their social status, and influence their behavior.

A few attempts were made to rebuild CMS but they did not result in changes at scale because of conflicts between government ministries. On the one hand the Ministry of Health hoped to use premiums to reduce the financial pressures on its health facilities and pay higher salaries. On the other, the Ministry of Agriculture did not want to burden farmers with additional fees. This conflict of interest was exacerbated because many rural health facilities were employing a significant number of largely untrained staff who had begun employment during the Cultural Revolution of the 1970s when training colleges were closed, and they needed to secure funding for their salaries and pensions. Once this group reached retirement age in the 2000s and rural facilities were able to recruit graduates of newly established medical colleges, there was a higher possibility that increased funding would provide benefits to rural residents. In addition, there was no clear agreement on the relative roles of different levels of government in financing health services. The limited local capacity of collecting fees and the low priority in the national reform agenda also contributed to the failed CMS rebuilding in the 1990s [50].

As public health care facilities and workers increasingly operated in a market, the strong, centralized, and hierarchical heath care administrative system played an important role in maintaining system resilience. However, as the former Rural Health Bureau Director Li Changming said “*The adaptive behavior of the public healthcare facilities and the health care workers under a market economy became increasingly difficult for the Ministry of Health to control*”.

There were two co-evolving patterns in the transition and adaption process: one was the health facilities and health workers who were becoming increasingly sophisticated participants in the health market often with support from the Ministry of Health; and the second was the efforts by the same ministry to maintain the basic health provision system. As one key informant Li Changming said during our interview “*we are health professionals and health officials, we had to make the health facilities function to serve the people*”.

### Second transition and adaptation: implementing the un-implementable through an experimentation process

The limited government funding of health care facilities led them to become increasingly reliant on charges to patients. Rural residents had to pay for treatment and medicines themselves, frequently resulting in impoverishment and/or foregoing of necessary treatment. This was generating pressure on the government to act.

The 2000 WHO report “*Health Systems: Improving Performance*”, ranking the fairness of financial contribution to health systems, ranked China at 188. “*This was considered a loss of face by top leaders*”, said by the former Health Minister Zhang Wenkang. In addition, “*the top leaders were shocked by the rural health conditions and the impoverishment of rural health care*” in China and decided to reform the rural health care system in 2002 and 2003. This was in the context of a change in the country’s political leaders and a shift in broad development policy in favor of taking active measures to ensure that all social groups benefited from China’s rapid economic development.

Faced with the difficulty of simultaneously modifying multiple components of the health system, the government decided on the development of NCMS as an entry point for reform. The new system was named New CMS (NCMS) in reference to the widely held view that the CMS had been an important achievement of health system development during the 1970s. Further, “*the many efforts to re-establish a social health financing scheme in rural areas during the 1990s provided very useful lessons for policy makers*”, Fu Wei, the NCSM office director said.

The landmark policy “*Decision on Further Strengthening the Public Health Work in Rural Areas*” was issued jointly by the Central Committee of the Communist Party of China and the State Council on October 19, 2002 [51]. The “Decision” made clear that rural China would establish NCMS, and the NCMS would be co-financed by the central and local governments and contributions from individuals. Central government would contribute 10 RMB, provincial and county governments were required to contribute 10 RMB, and individuals’ premiums were set as 10 RMB. The NCMS was designed to cover expenses from catastrophic illness and to be managed at county level instead of village and township levels.

### The basics for developing a functional health system

Several factors came together to provide a window of opportunity for the rapid development of NCMS. The Chinese government had made a political decision to alter its development strategy to ensure that all social groups benefited from economic growth. One aspect of this political change was a decision to make fiscal transfers to poor rural counties to support improvements in the provision of services. This was a major change in the management of public finance. The government was looking for “quick wins” to demonstrate its seriousness in meeting the needs of the rural poor.

Meanwhile, the Ministry of Health had supported a number of experiments with CMS, an organizational arrangement that would enable local governments to reimburse people for health costs they had incurred. A number of experienced experts were available to support the development of a national scheme. As a result, a mechanism was ready for translating the new government priority into practice. The government began by transferring very small amounts of money on a matching fund basis to CMS schemes, which reimbursed patients. These schemes were found to be effective in managing the money. Since the government wanted to transfer larger amounts of money, the existence of this mechanism enabled it to earmark the money for health on a matching funds basis. NCMS provided a functioning mechanism to transfer relatively large amounts of money with the confidence that a substantial proportion of the money would be paid to rural people as reimbursement for health care. “*This was an effective mechanism to ensure large numbers of people had visible benefits from this high profile government health program,*” Fu Wei said.

Crucially, in the early 2000s, medical facilities existed in rural areas all over China. Many had benefited from large government programs of investment in physical infrastructure, including health facilities. Also the hierarchical health management system was capable of negotiating with line ministries and local governments, providing a basis for the development of a functioning rural health system, indicated by the key informants at national and local levels.

One key aspect of the early phases of policy implementation was that pilot counties demonstrated a capacity to transfer money into a NCMS fund and to ensure that it was used to reimburse patients. The Ministry of Health put a lot of effort into creating an effective system for managing these flows of public funds [49]. In doing so, it demonstrated the possibility of subsidizing services used by residents of relatively poor areas [49, 52].

### Experimentation with incentives

With limited and variable capacity in county health administrations charged with implementing the NCMS, a lack of local-level data on burden of disease and health service utilization, and reliance on a huge number of implementing units, China adopted a experimentation process to allow local governments to adapt the scheme to local conditions and produce lessons that could contribute to scheme design and promote bottom-up learning in development of a central government policy [53, 54].

As with many Chinese policies, the central government set the parameters within which sub-national governments should work. Pilots were carried out in approximately 300 counties between 2003 and 2005. Many key elements of scheme design were left to local governments, including amounts of funding, insurance coverage, and design of reimbursement plans, and the management of funding and services. Expert teams were convened to help guide county-level pilots, develop training materials on NCMS design, and carry out training for local government officials and NCMS managers. In 2006, a large-scale interim evaluation of the scheme was carried out. This helped inform subsequent policy and promote convergence in policy design [54]. It was negotiated in the decision making period between government agencies, but once it was implemented, it became very simple as it was primarily the health ministry’s job, which injected money into the system and the Ministry of Health and health facilities started to build the provision again. As Fu Wei said, “*when funding is available, everyone is satisfied and the incentive remains strong*”.

### Expanding development investment to other components of the health system

Development of other parts of the rural health system, such as the management of medical facilities, drug procurement, establishment of Monitoring and Evaluation systems, and strengthening capacity in rural hospitals and clinics, came after the launch of the health insurance scheme. It is worth mentioning that in the entire process of NCMS development, the Ministry of Health made significant efforts to negotiate with the Ministry of Finance and the National Development and Reform Commission (NDRC), which are in charge of health facility investment to invest heavily in public health, township clinics, and village health stations, as well as county hospitals. The Ministry of Health also issued a series of policies to manage and improve the rural health workforce. The information system is also prioritized in the process of development [52].

### Changing the rules of the game and the compromise of system goals

The NCMS was initially a win-win for health facilities/health workers and the general population. Rural residents received tangible amounts of money as reimbursement for medical care. Health facilities generated income from the additional demand for services. Poor counties received a substantial amount of national and provincial money which more than matched their contribution. Over time, the facilities adapted to increase their share of resources. Further, there was competition between different facility levels, so each tried to benefit. Inevitably, this led to cost increases. The government responded with efforts to reform the health system and to ensure that a substantial share of the benefits went to the general public. However, it ran into major stakeholder interests, although the NCMS reform changed the rules of the game by providing substantial amounts of public finance and asking providers to think about the system goals^a^.

The evaluation of the NCMS came with the conclusion that the reform had achieved some success in financial protection of the catastrophic health care expenses, and the reform was retained [55–57]. The government rapidly increased the amount of money it contributed to these schemes.

### Third transition and adaption: rural health system under the National Healthcare Reform Agenda

In 2009, China embarked on an ambitious healthcare reform, with the goal of providing affordable and equitable basic health care for all by 2020, through universal health insurance coverage, establishing an essential drugs system, improving the primary health care delivery system, managing referrals to special care and hospitals, expanding public health services, and reforming public hospitals [58]. The rural health care reform decision body was located in the NDRC with the Ministry of Health^b^ as a member. Reform and management of the rural health system became a part of the national health care reform agenda. NCMS-led rural health system development faced a complex management and policy environment. Nowadays, the county hospitals are public but the investment decisions, financial power, and personnel managements are dispersed between many line ministries. The Ministry of Health and the NDRC are responsible for investment; the Ministry of Health, the Ministry of Finance, the Ministry of Human Resources and Social Security, and the Ministry of Civil Affairs are responsible for financial power; the Ministry of Health, the Ministry of Human Resources and Social Security, and the Party’s Organizational Department are in charge of personnel management [56].

After three years’ of reform effort the complexity of the change process has become increasingly clear. The government introduced an essential drugs policy to control costs and this was met with strong resistance from the doctors and the health care facilities [41]; the reform of county hospitals faced big challenges [59], and the implementation of reform is now understood to be a complex and challenging process [56]. While outside forces were exerting pressure on the delivery system (through financing system-payment reforms, through reforms in public county hospitals, through drug selling controls, through the creation of a competitive health providers’ game, etc.), agents actively adapted to each change in the rules to protect their interests. There is also intense stakeholder lobbying concerning any changes in the roles. For example, the Ministry of Finance has had difficulty in pushing through a reform agenda to lower costs, increase access to quality care when the health system stakeholders are adamant to protect their own interests^c^.

Because the national health care reform has been slow to show signs of success, the 2012 National Economic Conference did not include health care reform in its priority list [60]. In March 2013, the National Healthcare Reform Office was relocated to the newly formed National Health and Family Planning Commission. This has been taken by some analysts to be a sign that the health care reform agenda has less priority than before [61]. The third transition and adaption process is far from complete.

## Discussion

The case of NCMS provides useful insights into health system development in low- and middle-income countries. Each transition was triggered by a change in the national development agenda. When the macro-policy environment changed, its influence on the health system could be negative (the case of CMS collapse) or positive (the establishment of NCMS). Keeping the health agenda high in the development core agenda is essential to ensure that forced transitions result in beneficial health system development.

China’s rural health system has shown great resilience when facing outside forces because it has a strong, centralized, and hierarchical administrative system and the healthcare facilities are publicly owned. The Ministry of Health played a strong advocacy role in pushing for investment in facilities. It also promoted experimentation with an effective mechanism for managing fiscal transfer earmarked for health. The existence of a strong Ministry of Health has been an important force for resilience.

The Ministry of Health has had to balance its responsibilities for meeting national health system objectives with its role as an advocate for the interests of health service providers. It adopted various approaches to cope with the impact of the market economic reform by allowing health care workers and organizations to charge service fees without fully understanding the long-term impact of the emerging agent behavior on the system goals. These emergent behaviors created policy resistance or mal-adaption when the goal oriented new reform policies were introduced. In some cases, public ownership of hospitals and clinics has reinforced the behavior of agents as they use their position to reduce competition from private providers. This kind of interest group behavior could put the system at risk if it cannot deliver the expected performance to the public and to the government.

The third insight is the need to adapt implementation of policies to local contexts. China’s rural health system development between 2003 and 2008 shows that incentive structure is crucial and the limited and varying sub-national capacity need not be a barrier to developing functioning systems. Capable and motivated local government, health care officials, health organizations, and doctors are important for effective implementation. The national policies must be designed to allow flexible local management to get the implementation process started and, in the process, manage the convergence of the policy design.

Finally, the study suggests that an awareness of CAS concepts for understanding system behavior can provide a useful tool for analyzing the likely response to different policy interventions. The many problems that have arisen with the implementation of the rural health system reform policy illustrate the complex, political nature of the management of this kind of change. In particular, the case study highlights the important role of government in establishing clear health system objectives and in providing overall leadership for the management of system change.

China and many other countries are likely to experience rapid and interconnected changes for many years to come. Their health systems will need to adapt to these changes and to their impact on broad development policies. It will become increasingly important that the government increase its capacity to manage this kind of complex change process in order to create a resilient health system.

### Endnotes

^a^Discussions in national health care reform meetings where the chief author is a member*.*

^b^Ministry of Health, renamed National Health and Family Planning Commission since March, 2013*.*

^c^Internal discussions participated by the first author in the national health care reform meetings*.*

## Abbreviations

CAS: Complex adaptive systems
CMS: Cooperative Medical System
NCMS: New cooperative medical scheme
NDRC: National development and reform commission.
